# Editorial: Enhancing resilience in military personnel: insights into physiological, physical, psychological dimensions

**DOI:** 10.3389/fphys.2026.1783839

**Published:** 2026-02-20

**Authors:** Tommi Ojanen, Stefan Sammito, Olaf Binsch

**Affiliations:** 1 Human Performance Division, Finnish Defence Research Agency, Riihimäki, Finland; 2 Neuromuscular Research Center, Faculty of Sport and Health Sciences, University of Jyväskylä, Jyväskylä, Finland; 3 German Air Force Centre of Aerospace Medicine, Cologne, Germany; 4 Occupational Medicine, Faculty of Medicine, Otto-von-Guericke-University of Magdeburg, Magdeburg, Germany; 5 Human Perfomance Department, Netherlands Organisation for Applied Scientific Research, Soesterberg, Netherlands; 6 Netherlands Ministry of Defence, Military Health Department, Section for International Collaboration and Innovation, Utrecht, Netherlands

**Keywords:** augmentation, cognition, human performance, neurofeedback, nutrition, virtual reality

The Research Topic of “*Enhancing Resilience in Military Personnel: Insights into Physiological, Physical, Psychological Dimensions*” was launched in May 2024 as a part of NATO’s Science and Technology Organisations’ activity guided by its Human Factors and Medicine (HFM) Panel and executed by the Research Task Group 365 which focused on ‘Human Capability and Survivability Enhancement: *Aug*menting people to deliver an enhanced and more resilient capability for defence’. Enhancing resilience can be divided into psychological, physiological, and cognitive domains to give military personnel better understanding of HPA and its possibilities when planning and preparing for crisis and emergencies (Ziehr and Merkt, 2024; Casey et al., 2025).

The purpose of this Research Topic was to explore various aspects of human performance augmentation (HPA) technologies in military contexts. The physiological and psychological demands on military personnel have intensified in recent years due to greater complexity of modern military systems and to the increasing multiplicity in multi-domain operations on land, air, sea, and the cognitive- and in cyber domain. HPA involves the use of technological means to improve human performance above normal levels, restore and sustain health, and alter physiological functions (Ryan et al., 2023). Closing the gap between human performance augmentation research and training in the field allows for an improved coordination of efforts to better enhance and prepare soldiers (Billing et al., 2021). Performance can be monitored and predicted by using wearables to measure various factors, like duration and intensity of exposure to stressful events and sleep deprivation (by, e.g., heart rate, heart rate variability, blood/saliva markers testosterone, cortisol, immunological markers, respectively), physical load, and physical activity (e.g., load weight, accelerometry). However, the best and most efficient way to monitor and predict soldier performance still remains unfound. Most of the measures cannot provide immediate answers because of long analysis times, and also more research is needed to ensure sufficient accuracy of predicted outcomes (Main et al., 2025; Doyle et al., 2025; Kyröläinen et al., 2018).

This interdisciplinary Research Topic aimed to foster knowledge exchange and collaboration to advance the development of HPA among soldiers and to provide a unique opportunity to the experts in the areas of physiology, psychology, sport sciences, pharmacology, endocrinology, and medicine to present their work to the research community. Altogether eleven articles were accepted into this Research Topic. Themes of the articles included how human performance, nutrition, cognition, neurofeedback, and virtual reality could improve and/or enhance human performance.

In the study by Brunyé et al. 387 U.S. Army soldiers were studied to find links between the traits of military personnel and their performance outcomes. Among soldiers, traits like resilience, grit, emotion regulation, and mindfulness predicted outcomes across move, shoot, communicate, navigate, and sustain tasks under stress. The study showed that predicting human performance outcomes is challenging even with a diverse assessment battery, but future wearable biosensing technology could prove valuable for performance evaluation and prediction by combining physical, psychological and operational perspectives to understand human performance optimization better in an operational environment. Findings also support more targeted selection and training to enhance operational effectiveness.

The review by Jacques et al. explored the potential of neurofeedback training (NFT) to enhance cognitive performance and treat post-traumatic stress disorder in military personnel. It highlighted NFT’s applications before, during, and after deployment, and discussed its role among performance enhancement techniques. Key factors affecting NFT effectiveness and future research directions are outlined to guide military use and implementation.

In their study, Bottenheft et al. studied 35 Special Operations Forces (SOF) operators and found that 2 × 2 min cervical transcutaneous vagus nerve stimulation (ctVNS) did not improve cognitive or virtual reality-based operational performance under sleep deprivation. While sleep loss impaired the execution of all tasks conducted by the SOF operators, ctVNS showed no significant benefit. Higher stimulation intensity may negatively affect performance, suggesting dose optimization is the key. Further research is needed to refine protocols.

A review paper by Smid et al. explored how nutrition can support military performance in cold environments by mitigating cold-weather impacts on physiological and cognitive functions. Key strategies include preventing micronutrient deficiencies, enhancing vasodilation, and using stimulants like caffeine and tyrosine. While some promising interventions are identified, further research is needed to confirm their effectiveness and guide practical implementation.

Fallowfield et al. discussed in their mini-review about the whole system approach to improve military health and performance, addressing high injury rates and declining readiness. It critiqued individual-focused efforts and emphasized systemic, scalable interventions. Integrating leadership, strategy, and resource allocation is the key. Financial justification and system-based thinking are needed to enhance resilience, optimize human performance, and sustain operational effectiveness.

Cognition was studied by Zanesco et al. in their research, where lab-based attentional control tasks predicted performance in augmented reality military drills. Among 356 U.S. Army soldiers, better sustained attention and working memory were linked to higher accuracy in operational tasks, including shoot/don’t-shoot scenarios. These findings suggested that such cognitive tests can support evaluation, selection, and training to improve military readiness.

A perspective by de Vries et al. proposed an integrated system for monitoring military health and readiness using wearable tech, tailored indicators, and advanced tools like large-language-models (LLMs) and knowledge graphs. Article evaluated fragmented current methods and emphasized the need for comprehensive, task-specific insights to support performance, predict readiness, and manage long-term health risks in military personnel.

Human performance of elite military divers (Kelly et al.) was profiled using physical, physiological, and hormonal metrics. Divers showed high fitness levels and lean body composition. Strong links were found between physical performance (e.g., strength and explosive power) and hormone levels. Over time, testosterone and dehydroepiandrosterone levels shifted significantly during training, while cortisol remained stable. These findings highlight key stress and performance biomarkers in elite military training.

Koedijk et al. tested a Virtual Reality-based method to assess “action intelligence” in Dutch SOF personnel during close-quarters battle scenarios under conditions of sleep deprivation. SOF operators outperformed SOF support units in situational awareness and decision-making. While valid for some measures, visual response time proved unreliable. The test also showed no effect of sleep deprivation. Future refinements should focus on digitalization, team performance, and measurement accuracy.

In their review Feigel et al. highlighted that the allostatic load model as a promising framework for understanding how chronic stress during military training impacts long-term health. It emphasizes the need for a holistic view of warfighter health, integrating physical, psychological, and musculoskeletal domains. Wearable technology may help quantify stress-related risks, enabling early prevention of injuries and performance decline in military personnel.

Kjærgaard et al. used the Intervention Mapping framework in their research to construct and develop a military mental health training program in the Danish armed forces. The goal of their program was to promote mental health and operational readiness through their theory-driven approach, which included a systematic six-step process. This approach can offer a replicable roadmap for other military organizations, although more research is needed to evaluate long-term success in real-world military environment.

Articles in this Research Topic increased our understanding of HPA in military context. It also aimed to close the gap between cutting-edge research and operational training, ensuring that scientific innovation directly informs the preparation and support of soldiers. By fostering interdisciplinary collaboration across physiology, psychology, sport sciences, pharmacology, endocrinology, and military medicine, the Research Topic created a rare and important platform for advancing the science and application of HPA in military contexts. As indicated in the articles, already now and especially in future technologies (wearables, Virtual/Augmented/Mixed Reality, nerve stimulation, neurofeedback training), and well-structured training programs (physical and psychological) can provide effective new tools to augment Human Performance.

Unfortunately, the research on HPA that here was presented has largely overlooked neuroergonomics and biotechnological aspects, as well as their broader implications. This gap should be explicitly addressed in future studies. While several potential HPA tools already exist, armed forces must carefully assess which methods align with their specific needs and objectives. Such assessments should consider national legal frameworks, societal expectations, ethical considerations, and available resources. In particular, these perspectives must address challenges related to data privacy and external visibility, and consider whether informed consent can provide a viable safeguard. Finally, attention must be paid to dual-use concerns, as many of these technologies are primarily developed in the civilian domain, as well as their practical and ethical implications when applied in the military domain.
